# Combination of Fusiform Capsulectomy of the Posterior Capsule and Percutaneous Flexion Tendon Release in the Treatment of Fused Knee with Severe Flexion Contracture During Total Knee Arthroplasty—A Report of Six Cases

**DOI:** 10.3389/fsurg.2022.859426

**Published:** 2022-05-23

**Authors:** Qun-Qun Chen, Min-Cong He, Zheng Cao, Xiang-Peng Kong, Hai-Bin Wang, Wei Chai

**Affiliations:** ^1^ Department of Orthopaedic Surgery, The Third Affiliated Hospital of Guangzhou University of Chinese Medicine, Guangzhou, China; ^2^ Guangdong Research Institute for Orthopedics and Traumatology of Chinese Medicine, Guangzhou, China; ^3^ Department of Orthopedics, The Fourth Medical Center of PLA General Hospital, Beijing, China; ^4^ School of Medicine, Nankai University, Tianjin, China; ^5^ National Clinical Research Center for Orthopedics, Sports Medical and Rehabilitation, Beijing, China; ^6^ Department of Orthopaedic Surgery, The First Affiliated Hospital of Guangzhou University of Chinese Medicine, Guangzhou, China

**Keywords:** total knee arthroplasty, fused knee with severe flexion contracture, fusiform capsulectomy of posterior capsule, percutaneous flexion tendon release, functional score

## Abstract

**Purpose:**

This clinical research aims to assess the safety and efficacy of a combination of fusiform capsulectomy of the posterior capsule and percutaneous flexion tendon release in the treatment of a fused knee with severe flexion contracture during total knee arthroplasty (TKA).

**Methods:**

A retrospective analysis was performed in three patients (six knees) who had preoperative severe bony fused flexion contracture (>80°) prior to TKA and received a combination of fusiform capsulectomy of posterior capsule and percutaneous flexion tendon release during TKA between January 2016 and December 2019. The range of motion (ROM), knee functional score, postoperative complications, and radiographic results were evaluated.

**Result:**

Three patients (six knees) were enrolled in this study. The mean duration of follow-up was 42.83 ± 15.77 months. The postoperative knee ROM was 100.0 (76.0, 102.75) (*p *< 0.01). The knee society score (KSS) clinical score increased from a preoperative 30.0 (25.0, 36.0) to a postoperative 64.0 (65.0, 78.0) (*p *< 0.01), and the KSS function score increased from a preoperative 0.0 (0.0, 30.0) to a postoperative 55.0 (40.0, 55.0) (*p *< 0.01). No implant loosening, infection, neurovascular complications, or revision were recorded in the cohort until the last follow-up.

**Conclusion:**

The technique of a combination of fusiform capsulectomy of the posterior capsule and percutaneous flexion tendon release is an effective and safe method during primary TKA for a fused knee with severe flexion contracture.

## Introduction

Conversion of a severely fused knee to a total knee arthroplasty (TKA) is a challenging procedure with many complications. High incidence rates of various complications (53%–57%) have been reported, such as residual extensor lag and postoperative stiffness (1, 2). The routine techniques for correcting flexion contracture include excessive osteotomy and soft tissue release. However, excessive osteotomy usually cannot repair a severely fused knee in flexion effectively and restore stability without damaging the collateral ligaments and elevating the joint line (3). Posterior capsular release, combined with distal femur resection up to a maximum of 4 mm, has been proven to be effective in the treatment of flexion contracture, but only 5° correction has been achieved after medial soft tissue release and 2-mm additional osteotomy (3, 4). Residual flexion contracture will lead to an excessive requirement of quadriceps force in daily activities such as walking, which can cause aseptic loosening and unsatisfied clinical outcome. Previously, we had introduced a technique of fusiform capsulectomy of the posterior capsule to correct severe flexion contracture during primary TKA (5). In this study, for those with a fused knee with severe flexion contracture and that cannot be corrected by fusiform capsulectomy, we prescribe a combination of fusiform capsulectomy of the posterior capsule and percutaneous flexion tendon release and evaluate the clinical efficacy and safety.

## Materials and Methods

We retrospectively analyzed patients who had a fused knee with severe flexion contracture (>30°) and received a combination of fusiform capsulectomy of the posterior capsule and percutaneous flexion tendon release during primary TKA between January 2016 and December 2019. The inclusion criteria were as follows: (1) those whose preoperative fused knee with severe flexion contracture was more than 30°; (2) all surgeries were performed by one surgeon (C.W.); and (3) the follow-up period was more than 12 months. The exclusion criteria were as follows: (1) those who had missed providing necessary clinical data and (2) those who failed follow-up treatment regularly. This study was approved by our institutional review board (S2018-018-01), and written informed consent of all patients for data analysis was obtained retrospectively.

The preoperative and postoperative clinical evaluation were performed by two independent observers, including the preoperative and postoperative ranges of motion, knee society score (KSS) clinical score and KSS functional score, and perioperative and postoperative complications. The radiographs of the knee and lower extremities were used to determine the component position changes, limb alignment, and evidence of loosening or osteolysis.

### Surgical Procedure

**Figure 1:**
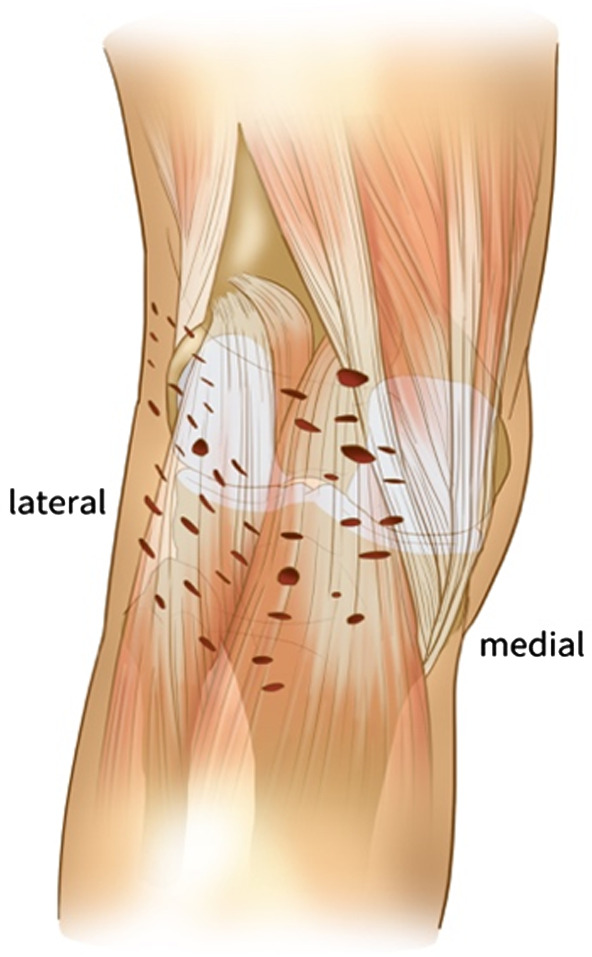
Schematic diagram of the percutaneous flexion tendon release of a severe fused knee.

**Figure 2:**
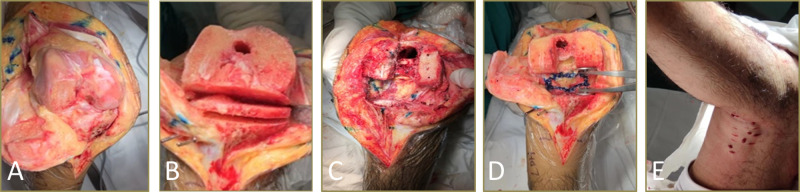
Surgical technique. (**A**) The joint cavity was exposed using the quadriceps oblique cutting technique. (**B**) In the knee flexion position, the 10-mm standard osteotomy was performed in the distal femur, and then the knee joint cavity was opened through the “double knife method” at the height of the joint line. (**C**,**D**) The fusiform capsulectomy of the posterior capsule was used to loosen the posterior joint capsule of the knee. (**E**) Percutaneous flexion tendon release was done to loosen the posterior joint capsule of the knee joint.

All patients had general anesthesia and pneumatic thigh tourniquets. Cemented knee prosthesis was adopted in all patients. The knees were exposed through the standardized medial parapatellar approach. After removing the osteophytes and releasing soft tissue routinely, the quadriceps oblique cutting technique would be used to expose the joint cavity. In the knee flexion position, the 10-mm standard osteotomy was performed in the distal femur, followed by opening the knee joint cavity through the “double knife method” at the height of the joint line. The first knife was at the joint line height of the knee flexion position, and the second knife was within 10 cm below the joint line. After completing the above operations, the spacer block was used to evaluate the extension gap. If the extension gap was significantly less than 20 mm, additional osteotomy in the distal femur was performed, and the osteotomy should not be more than 4 mm to avoid injury to the attachment of the medial and lateral collateral ligaments of the knee on the femoral side. If the extension gap was still not enough, the fusiform capsulectomy of posterior capsule would be performed. First, a spreader was used to open the flexion gap and mark the resection range in a 90° inflection of the knee. The resection margins were limited to the medial and lateral collateral ligaments, the posterior femoral condyles, and the tibial plateau to avoid injuring the popliteal vessels and the common peroneal nerve. Second, the posterior capsule was grasped with Kocher or forceps and the posterior capsule was resected with a lower output electrosurgical knife. The electrosurgical knife was controlled below 30 W, and the sleeve was used to limit the cutting depth within 2–3 mm. The fusiform capsulectomy of the posterior capsule should be completed until the fat or muscle tissue is found behind the posterior joint capsule. During the procedure of fusiform capsulectomy of the posterior capsule, the neurovascular bundle is generally not visible. This operation is very superficial, and only a part of the joint capsule is removed to release the knee joint. Once the fat behind the posterior joint capsule is found, great care must be taken. After the above procedures, the spacer block was used again to check the flexion and extension gap. Generally, the flexion contracture could be corrected by at least two-thirds for patients with ankylosing spondylitis (AS). According to the proper size and rotation of the distal femur and proximal tibia, the other procedures of osteotomy were performed. The tightest flexion tendon at the back of the knee joint was located in extension position, following with percutaneous flexion tendons releasing behind the knee joint with a sharp knife. The released part was mainly the tendon of semitendinosus, semimembranosus, and biceps femoris, and the depth of release was controlled within 5 mm. Multiple parts of release were performed above and below the tendons as necessary until satisfactory straightening was achieved (Figures 1, 2). Finally, the cemented knee prosthesis was implanted.

All patients were given 2.0 g of ceftriaxone intravenous antibiotics once a day for a postoperative period of 48 h and 100 mg of aspirin once a day postoperatively for 2 weeks. Among the patients included in the study, residual flexion contracture was corrected by wearing an extended plaster splint and pressing the weight of 10 pounds for 12 weeks. Contracture deformity was fixed at night by using a plaster splint in the maximum extended position. The weight was lifted above the knee during the day (5–10 kg) to improve the knee extension.

### Statistical Analysis

Statistical analysis was performed with SPSS software 22.0 (SPSS, Chicago, IL, USA). Data were presented as means ± standard deviation or median with interquartile. The differences between the groups were assessed by using the paired *t*-test. A *p-*value of <0.05 (two-tailed) was considered significant.

## Results

From January 2016 and December 2019, we performed the technique of fusiform capsulectomy of the posterior capsule in three patients (six knees) during knee replacement. All of them were successfully followed up and enrolled in the study. Preoperative clinical information of three patients (six knees) is given in Table 1.

**Table 1:** Patient demographics.

Patients	Gender	Age	Side	BMI (kg/m^2^)	Cause of ankylosis	Range of motion	Degree of flexion ankylosis
1	Male	35	L	29.8	AS	0	95
			R			0	95
2	Male	27	L	27.7	AS	0	80
			R			0	90
3	Male	32	L	19.5	AS	0	95
			R			0	95

*M, male; F, female; L, left; R, right; AS, ankylosing spondylitis.*

The prostheses in this study were PFC sigma (two knees) and TC3 (four knees) (DePuy, Warsaw, IN, USA). The average follow-up was 42.83 ± 15.77 months (range, 28–63 months). The exact pre- and postoperative range of motion (ROM) of every knee at every time point is given in Table 2 and Table S1. In the last follow-up, the postoperative knee ROM was 100.0 (76.0, 102.75) (*p* < 0.01). The KSS clinical score increased from a preoperative 30.0 (25.0, 36.0) to a postoperative 64.0 (65.0, 78.0) (*p* < 0.01), and the KSS function score increased from a preoperative 0.0 (0.0, 30.0) to a postoperative 55.0 (40.0, 55.0) (*p *< 0.01) (Table 2).

**Table 2:** Results of total knee arthroplasty in patients with flexion ankylosis of the knee after final follow-up.

Patients	Follow-up time (months)	Side	Range of motion					KSS function score		KSS clinical score		Complications
			Preop	Postop								
				6 ms Postop (°)	12 ms Postop (°)	24 ms Postop (°)	Last F/U (°)	Preop	Postop	Preop	Postop	
1	38	L	0	5–100	5–100	5–105	5–105	30	55	36	65	−
	37	R	0	5–100	5–102	5–102	7–102	30	55	36	65	−
2	63	L	0	3–60	3–65	3–64	3–64	0	55	25	52	−
	62	R	0	4–85	4–80	4–80	4–80	0	55	25	76	−
3	29	L	0	8–100	10–100	10–100	10–100	0	40	30	78	−
	28	R	0	5–104	10–100	10–100	10–100	0	40	30	78	−

*F/U, follow-up; Preop, preoperative; Postop, postoperative; KSS, knee society score.*

### Typical Case

**Figure 3:**
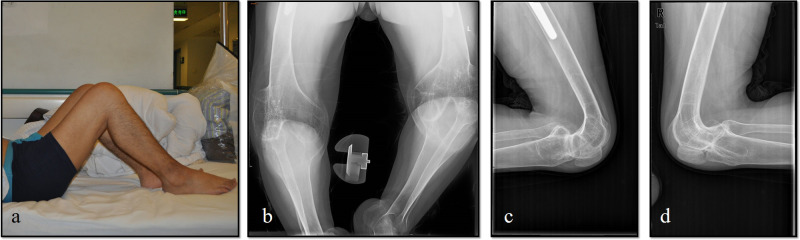
Patient with a severe bony knee flexion contracture before surgery and was not able to walk. (**a**) Preoperative appearance of a 38-year-old female with AS showing osseous ankylosis of both knees. (**b–d**) Preoperative Anterior-Posterior and lateral views of both knee showed osseous ankylosis fixed at degree of 95° without range of motion.

**Figure 4:**
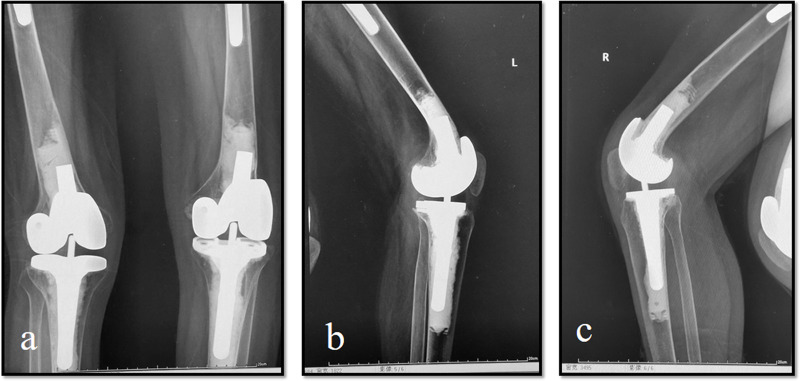
The range of motion and bony flexion contracture significantly improved after surgery. (**a**) Postoperative radiographs following bilateral total knee arthroplasty at 12 months. (**b**) Left knee can be flexed to 100° postoperatively. (c) Right knee can be flexed to 102° postoperatively.

Male, AS, and bilateral TKA preoperative bony flexion contracture angles were 95° in both knees (Figure 3). A combination of fusiform capsulectomy of the posterior capsule and percutaneous flexion tendon release was used. Plaster splint was applied at night to correct the residual contracture deformity. The weight of 5–10 kg was lifted above the knee. The postoperative compression was fixed for 3–4 weeks. The ROMs of both knees significantly improved after surgery (Figure 4).

## Discussion

In this study, there are promising clinical and radiological results after TKA for patients with severe bony ankylosed knees in flexion with a combination of fusiform capsulectomy of the posterior capsule and percutaneous flexion tendon release.

Multiple pathological causes can induce bony ankylosed knee joints, including trauma, AS, rheumatoid arthritis, and septic disease (6). Arthrodesis, an optional treatment for correcting ankyloses of the knee, can provide a stable and painless knee joint, but the function is poor (7). Total joint replacement for young patients is controversial because of the expected duration and wear of prosthetic, as well as the fatigue of materials in certain long period.

The reason for the bony ankylosed knee is complicated. In the early stage of inflammation (especially in AS or rheumatoid arthritis patients), patients may inconsciently settle the knee in flexion position because the pain caused by joint swelling can be partially released with a larger capacity of the knee joints. The posterior joint capsule is thickened and fibrosis is accompanied by a contraction of the flexion tendon and, finally, the knee joint is fixed with bony fusion in flexion position (8).

To correct the bony deformity of the knee in flexion position, one-stage or two-stage surgery is considered. A separation of the fusion knee joint, followed by a skeletal traction of the distal tibia, is required before TKA. A proper soft tissue release around the knee is often necessary. A complete correction of the flexion contracture can not only eliminate pain as much as possible but also provide a significant improvement in the quality of life of the patient (9, 10). Therefore, any effort to correct the deformity is essential during TKA. A common solution to deal with severe bony ankylosed knees in flexion is to increase the osteotomy of the distal femur. Its effectiveness and feasibility have been proved by many studies (4, 11, 12). However, excessive osteotomy can damage the collateral ligaments and weaken the quadriceps muscle. In order to avoid the elevation of the joint level, the maximal range of distal femur cutting was kept at 14 mm in this study. Soft tissue release was needed to correct the resident deformity after judicious bone osteotomy. As mentioned above, soft tissue release, particularly for the posterior soft tissue, plays a crucial role in improving contracture. Selective capsulotomy was reported in a previous study. Taylor et al. reported that posterior capsule incision is effective in the treatment of the knee flexion contracture in children with cerebral palsy (13). Masuda’s study describes that posteromedial vertical capsulotomy is an effective method to increase the extension gap and achieve gap balance during TKA (14). The percutaneous pie-crusting technique was proved to be effective in correcting the quadriceps tendon contracture in extension stiffness knees (15, 16). Our previous study introduced a safe and effective fusiform capsulectomy technique that could correct severe flexion contracture during primary TKA (5). Rather than simply incision or vertical capsulotomy of the posterior capsule, fusiform capsulectomy can enlarge the articular cavity and effectively open the posterior gap. The resection depth should be limited to 2–3 mm to avoid the popliteal vessels and prevent nerve injuries.

Most of the patients can realize a satisfying extension ROM during surgery, but the long-time bony fusion may induce not only the posterior capsule contracture but also other soft tissue behind the contracture knee joint shortening, including the gastrocnemius muscles. Scarring of the flexion mechanism with fibrosis and shortening of the gastrocnemius muscles contribute partially to the flexion contracture. To fully correct the contracture joint, we performed a percutaneous flexion tendon release with the percutaneous pie-crusting technique. No. 11 surgical blade with a limited depth of 5 mm was used in the flexion tendon release. This minimally invasive technique can further improve the clinical results and acquire no major complications. Postoperative compression and straight splint were used to eliminate the potential risk of recurrence of postoperative flexion contracture.

Several limitations existed in this study. First, the sample size was not large enough. Therefore, more cases with longer follow-up times need to be studied in the future. Second, this was a retrospective study, which could provide only limited clinical evidence. Lastly, a cadaveric study is needed to define the depth and range of the percutaneous pie-crusting skills.

## Conclusion

The technique of a combination of fusiform capsulectomy of the posterior capsule and percutaneous flexion tendon release to correct a fused knee with severe flexion contracture during primary TKA is both effective and safe.

## Data Availability

The original contributions presented in the study are included in the article/Supplementary Material; further inquiries can be directed to the corresponding author/s.
