# Supplementary material for: Combination of Fusiform Capsulectomy of the Posterior Capsule and Percutaneous Flexion Tendon Release in the Treatment of Fused Knee with Severe Flexion Contracture During Total Knee Arthroplasty—A Report of Six Cases
Source: Front Surg. 2022 May 23;9:859426. doi: 10.3389/fsurg.2022.859426 (PMC9407035; doi:10.3389/fsurg.2022.859426)
Supplement: Supplementary file 1 [file Table_1_v1.docx]

| Patients | Gender | Age | Side | BMI(kg/m2) | Cause of ankylosis | Range of Motion | Degree of flexion ankylosis |
| --- | --- | --- | --- | --- | --- | --- | --- |
| 1 | male | 35 | L | 29.8 | AS | 0 | 95 |
|  |  |  | R |  |  | 0 | 95 |
| 2 | male | 27 | L | 27.7 | AS | 0 | 80 |
|  |  |  | R |  |  | 0 | 90 |
| 3 | male | 32 | L | 19.5 | AS | 0 | 95 |
|  |  |  | R |  |  | 0 | 95 |

M: male; F: female; L: left; R: right; AS= ankylosing spondylitis
