# Supplementary material for: Combination of Fusiform Capsulectomy of the Posterior Capsule and Percutaneous Flexion Tendon Release in the Treatment of Fused Knee with Severe Flexion Contracture During Total Knee Arthroplasty—A Report of Six Cases
Source: Front Surg. 2022 May 23;9:859426. doi: 10.3389/fsurg.2022.859426 (PMC9407035; doi:10.3389/fsurg.2022.859426)
Supplement: Supplementary file 2 [file Table_2_v1.docx]

Table 2. Results of total knee arthroplasty in patients with flexion ankylosis of the knee after final follow-up

| Patients | Follow-up time (mons) | Side | Range of Motion | | | | | KSS Function Score | | KSS Clinical Score | | Complications |
| --- | --- | --- | --- | --- | --- | --- | --- | --- | --- | --- | --- | --- |
|  |  |  | Pre-op | Post-op | | | |  |  |  |  |  |
|  |  |  |  | 6ms Post-op | 12ms Post-op | 24ms Post-op | Last F/U | Preop | Postop | Preop | Postop |  |
| 1 | 38 | L | 0 | 5-100° | 5-100° | 5-105° | 5-105° | 30 | 55 | 36 | 65 | / |
|  | 37 | R | 0 | 5-100 | 5-102 | 5-102 | 7-102° | 30 | 55 | 36 | 65 | / |
| 2 | 63 | L | 0 | 3-60° | 3-65° | 3-64° | 3-64° | 0 | 55 | 25 | 52 | / |
|  | 62 | R | 0 | 4-85° | 4-80° | 4-80° | 4-80° | 0 | 55 | 25 | 76 | / |
| 3 | 29 | L | 0 | 8-100° | 10-100° | 10-100° | 10-100° | 0 | 40 | 30 | 78 | / |
|  | 28 | R | 0 | 5-104° | 10-100° | 10-100° | 10-100° | 0 | 40 | 30 | 78 | / |

F/U: follow-up; Preop: preoperative; Postop: postoperative; KS: Knee Society.
